# High-gain and low-profile antenna using novel reflective surface for satellite applications

**DOI:** 10.1371/journal.pone.0343506

**Published:** 2026-02-23

**Authors:** Lan Ngoc Nguyen

**Affiliations:** Wireless Communication Laboratory, Posts and Telecommunications Institute of Technology, Ho Chi Minh City, Vietnam; Gwangju Institute of Science and Technology, KOREA, REPUBLIC OF

## Abstract

A low-profile and compact antenna that offers wideband and high-gain features for satellite applications is investigated in this work. Instead of using an AMC surface for enhancing gain as in conventional antenna designs, this paper proposes a reflective surface made of pure copper laminate, and the results exhibit a 5 dB gain enhancement. In addition, the broadband performance is realized by loading partial ground technique. As a result, only four patch elements, the antenna designed at the central frequency of 4.3 GHz exhibits a bandwidth of 21.4% at −10 dB, a peak gain of 14 dBi and an efficiency over 80% over the frequency range of operating. The design concept is validated through measurements conducted on a fabricated prototype. In addition, the performance of the proposed antenna is compared with existing literature in order to highlight the importance of the proposed work.

## Introduction

Recently, due to the rapid advancement of satellite communication technology, the demand for high-capacity and high-speed communication is becoming increasingly important for the future [1–3]. To satisfy the above requirements, high-gain and broadband antennas are always required.

Besides, microstrip patch antennas are a popular choice in antenna design thanks to their features of low profile, low cost, and easy integration [4,5]. Numerous approaches have been explored to improve the performance of microstrip antennas, including gain enhancement [6–11] and bandwidth extension [12–19]. For the designs in [6] and [7], the authors proposed the solution of utilizing a substrate integrated waveguide (SIW) to enhance the performance of the antennas. As a result, the peak gain of these antennas achieves 9 dBi and 11 dBi. Similarly, the methods of differential-fed and sequential rotational phase feeding are adopted for the proposals in [8] and [9]. Incorporating grounded bars into a traditional square dielectric patch resonator not only shifts the higher-order TM_121_ mode to a higher frequency, thereby improving the antenna’s gain, but also facilitates the excitation of the TM_321_ mode, which can be combined with the TM_121_ mode to achieve a broader bandwidth in [8]. In [9], a 2 x 2 array employing the sequential rotational feeding is presented. The designs in [8] and [9] achieve a compact size and high gain.

Another gain enhancement approach proposed in [11] involves the use of an Artificial Magnetic Conductor (AMC) surface as a reflector. This technique improves gain and directivity by suppressing back and side lobes, thereby redirecting power toward the main lobe. The AMC’s structure and resonant frequency can be obtained by adjusting the dimensions of its unit cells. Experimental results in [11] demonstrate a bandwidth of 21%, a peak gain of 7.34 dBi, and a maximum efficiency of 71.5%. However, the designs in [6–10] still suffer narrow bandwidths below 8%, while the gain in [11], remains relatively low, not exceeding 8 dBi.

Related to bandwidth enhancement, multiple strategies have been suggested; for example: metasurface [12,13], cavity [14], defected ground structure (DGS) [15,16], parasitic elements [17,18], AMC surface [19]. By employing characteristic modes analysis (CMA) to customize a non-uniform metasurface and exploiting as a superstrate, the design in [12] exhibits a substantial improvement in bandwidth, which is also observed in [13]. Meanwhile, with the aid of cavity [14], DGS [15,16], and parasitic elements [17,18], and [19], higher-order resonances are generated, and as a result, the bandwidths of the proposals in these works are 48.3%, 13.37%, 10%, 28%, 20.8%, and 70%, respectively. Although the implementation of each method is different, they share the common principle of generating consecutive resonances. Nevertheless, these proposals are consistently characterized by low-gain radiation, remaining below 9 dBi. In contrast, other studies [20–22] demonstrate better performance in both gain and bandwidth, but the large size is a limitation of these studies. In general, simultaneously obtaining high gain, broad bandwidth, and compact size is still a demanding task.

In this work, a simple and effective approach is proposed for designing an antenna that offers both wideband, high-gain, and compact characteristics. Rather than employing an AMC surface commonly used in traditional antenna designs to enhance gain, this study proposes a reflector that is a pure copper laminate. The results reveal a gain improvement of 5 dB and this contributes considerably to the optimization of the antenna’s directivity and gain. Moreover, the presence of a partial ground technique results in enhanced broadband performance. These solutions are applied for the patch antenna array of 1 x 4 and the measured results indicate that with a total dimension of 2.33λ x 0.93λ x 0.098λ where λ is the free-space wavelength at the lowest operating frequency of 4 GHz yields an impedance bandwidth at -10dB of 21.4%, a peak gain of 14 dBi, a low sidelobe level, an efficiency exceeding 80% across the operating frequency range. In comparison with other reported designs, the proposed antenna offers the advantage of high gain while maintaining a low profile and utilizing only four elements.

## Antenna design and characteristics

### Design of an individual antenna element

**Fig 1:**
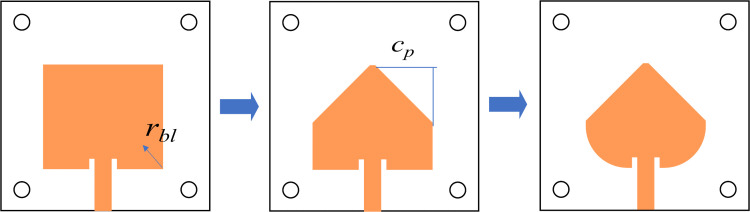
The design process of final geometry for the proposed antenna.

Firstly, the antenna design workflow is initiated by constructing the single element, and the overall design process is illustrated in Fig 1. The initial radiator was selected as a planar microstrip patch antenna owing to its low profile, ease of fabrication, and good compatibility with printed feeding networks. A conventional rectangular microstrip patch fed by a microstrip line was first adopted as the baseline configuration. The geometric modifications were progressively introduced to enhance impedance matching and radiation performance. Firstly, the two upper corners of the rectangular patch were chamfered with a length denoted as *c*_*p*_ to mitigate abrupt current discontinuities at the radiating edges. Subsequently, the lower corners of the patch were truncated using circular arcs with radius *r*_*bl*_, resulting in a tapered transition region between the radiating patch and the feeding line.

**Fig 2:**
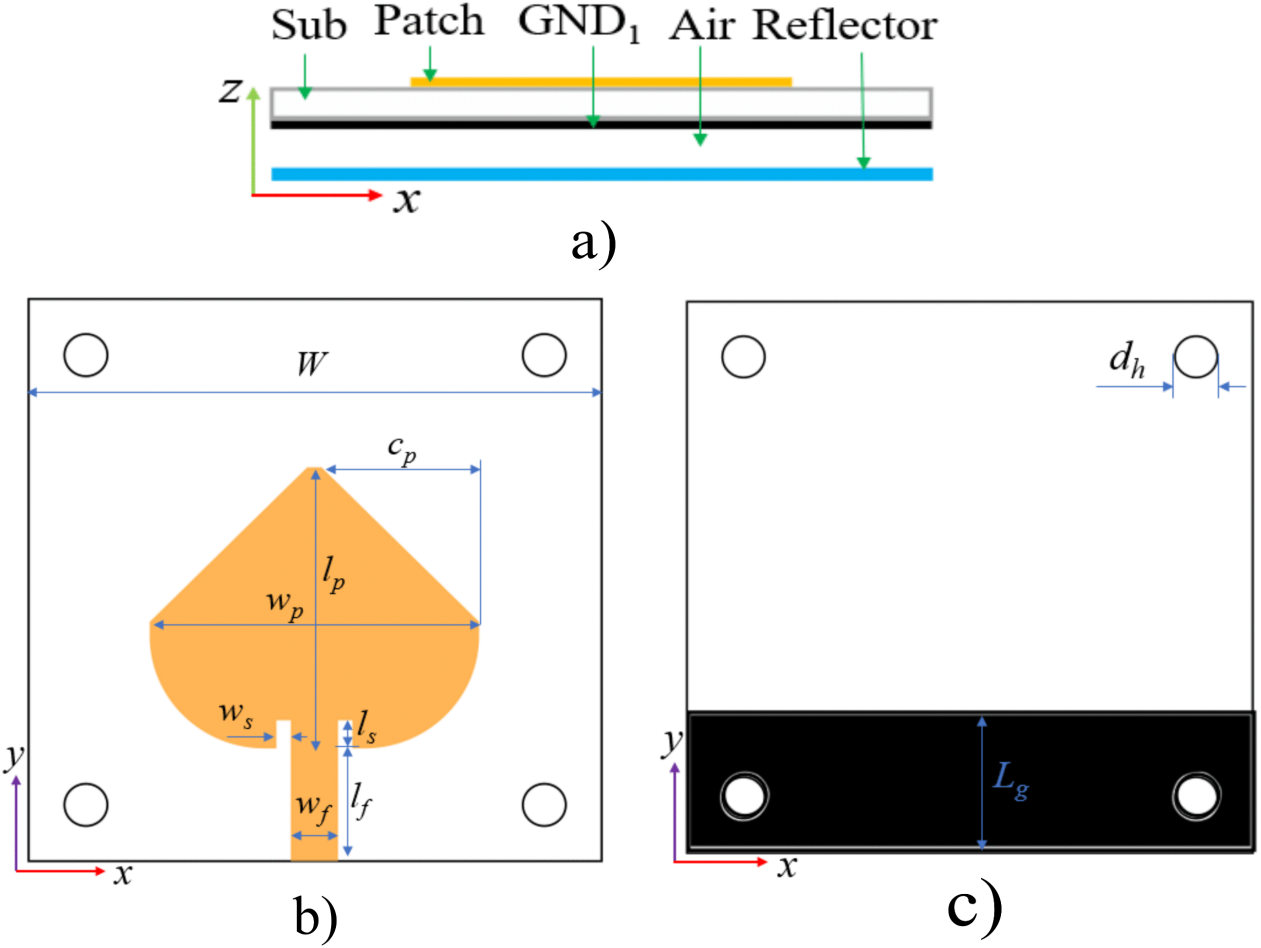
The geometry of the single element: (a) cross-section view; (b) the patch radiation element (left) and the partial ground (right). *W* = 40, *c*_*p*_ = 11, *w*_*p*_ = 23, *l*_*p*_ = 20, *w*_*s*_ = 1, *w*_*f*_ = 3.3, *L*_*g*_ = 10, *d*_*h*_ = 3, *l*_*s*_ = 2, *l*_*f*_ = 8, *h*_*a*_ = 5 (unit: mm).

The detailed antenna geometry corresponding to the aforementioned design procedure is illustrated in Fig 2. A patch as the principal radiating component and a partial ground layer are built on the top and bottom of a 1.524-mm-thick Roger RO4350B ($\varepsilon_{r}=\text{3}.\text{66},\delta =\text{0}.\text{0037}$), respectively. As aforementioned, the partial ground technique is employed to broaden antenna bandwidth; however, it also results in increased side and back lobe levels, which in turn reduce the antenna’s gain and directivity. To address this issue, a reflective surface, which is 0.8 mm thick copper laminate, is employed for aiming gain and directivity enhancement. Additionally, a 5 mm air gap (*h*_*a*_) is introduced between the dielectric layer and the copper laminate to improve bandwidth. The antenna is designed at the central frequency of 4.3 GHz for satellite applications [23]. The antenna has overall dimensions of 40 × 40 × 7.324 mm, with the patch measuring 23 × 20 mm, while the length of the ground layer is 10 mm. Multiple feeding configurations can be employed for microstrip antennas, including microstrip line [24], coaxial probe [25], aperture coupling [26], and proximity coupling [27]. In this paper, the microstrip-line feed is chosen because of its easy fabrication and simple impedance matching.

**Fig 3:**
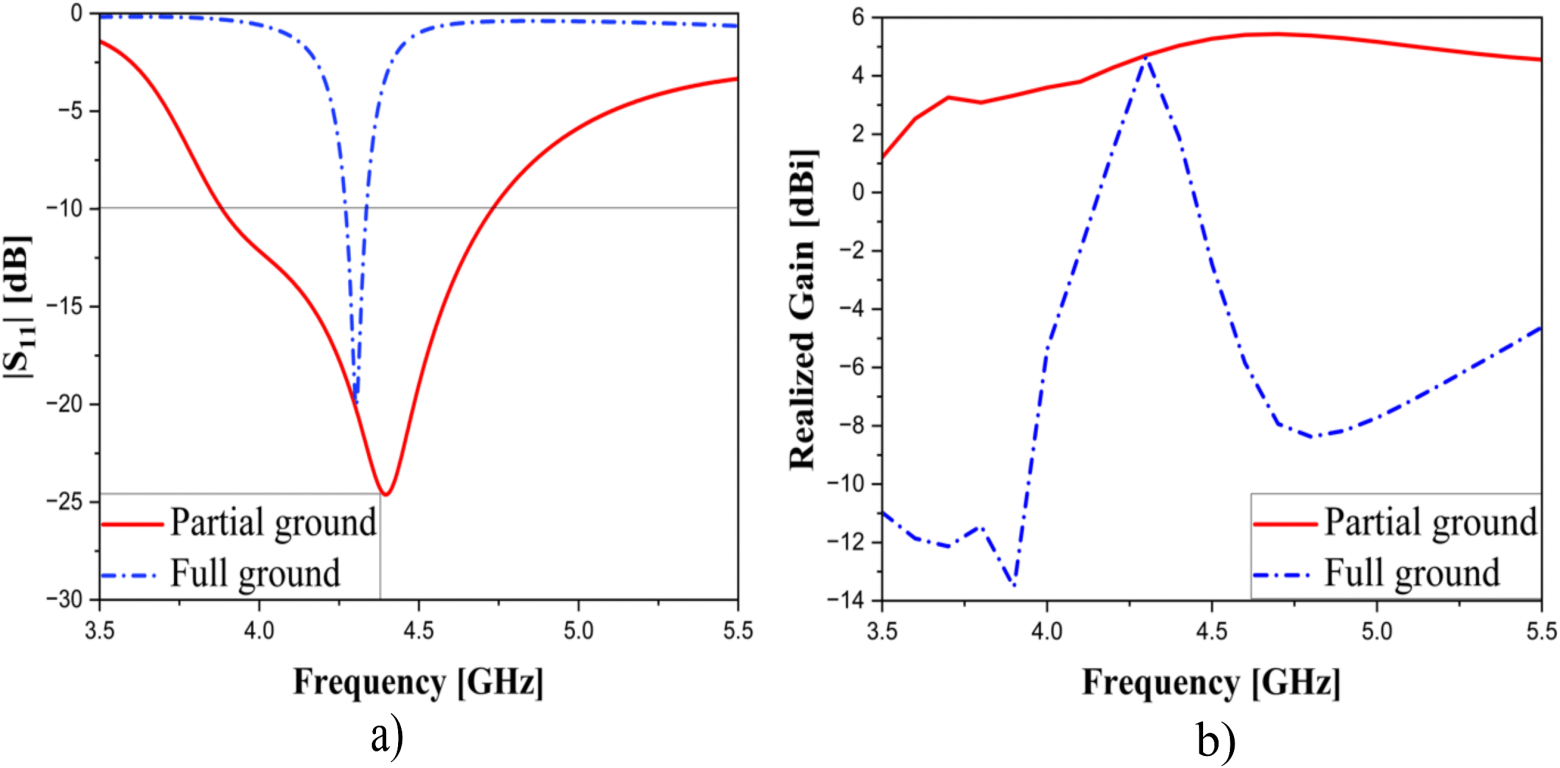
|S_11_| (left) and Realized Gain (right) with and without partial ground.

**Fig 4:**
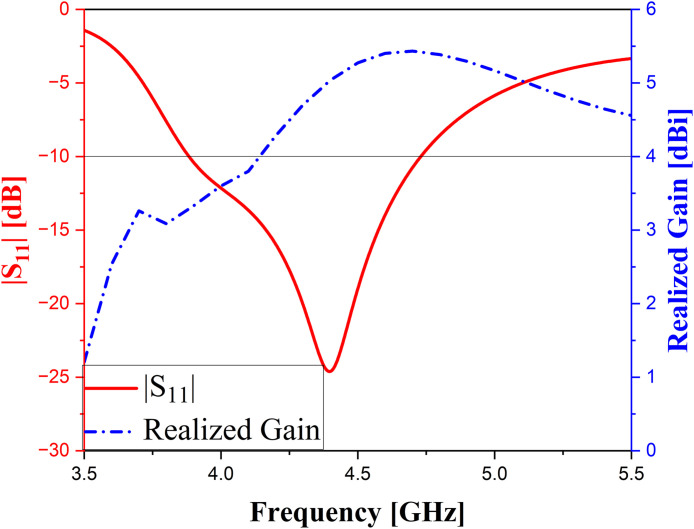
|S_11_| and Realized Gain of the single element.

Besides, to evaluate the effectiveness of the partial ground technique in enhancing bandwidth, a comparison in terms of performance is conducted between antennas with and without the proposed method, and this is depicted in Fig 3. It is evident that the change from full ground to partial ground not only enhances the antenna’s performance but also contributes to size reduction. This improvement is demonstrated by the increase in bandwidth from 66 MHz (1.5%) to 850 MHz (19.8%) and the gain from −13.54–4.67 dBi to 3.3–5.4 dBi (in frequency range of 3.9–4.7 GHz), respectively. Moreover, the depth of impedance matching is also improved. By utilizing a partial ground, parallel capacitances and inductances are introduced, and the analysis and calculation for inductances and capacitances are discussed in [28]. For this reason, the expansion of the operating frequency range is implemented. Fig 4 shows the reflection coefficient and realized gain of the single element. As observed, the bandwidth of the antenna achieves 19.8% and the gain value fluctuates from 3.33–5.43 dBi in the operating range of frequency.

### Design of an array

**Fig 5:**
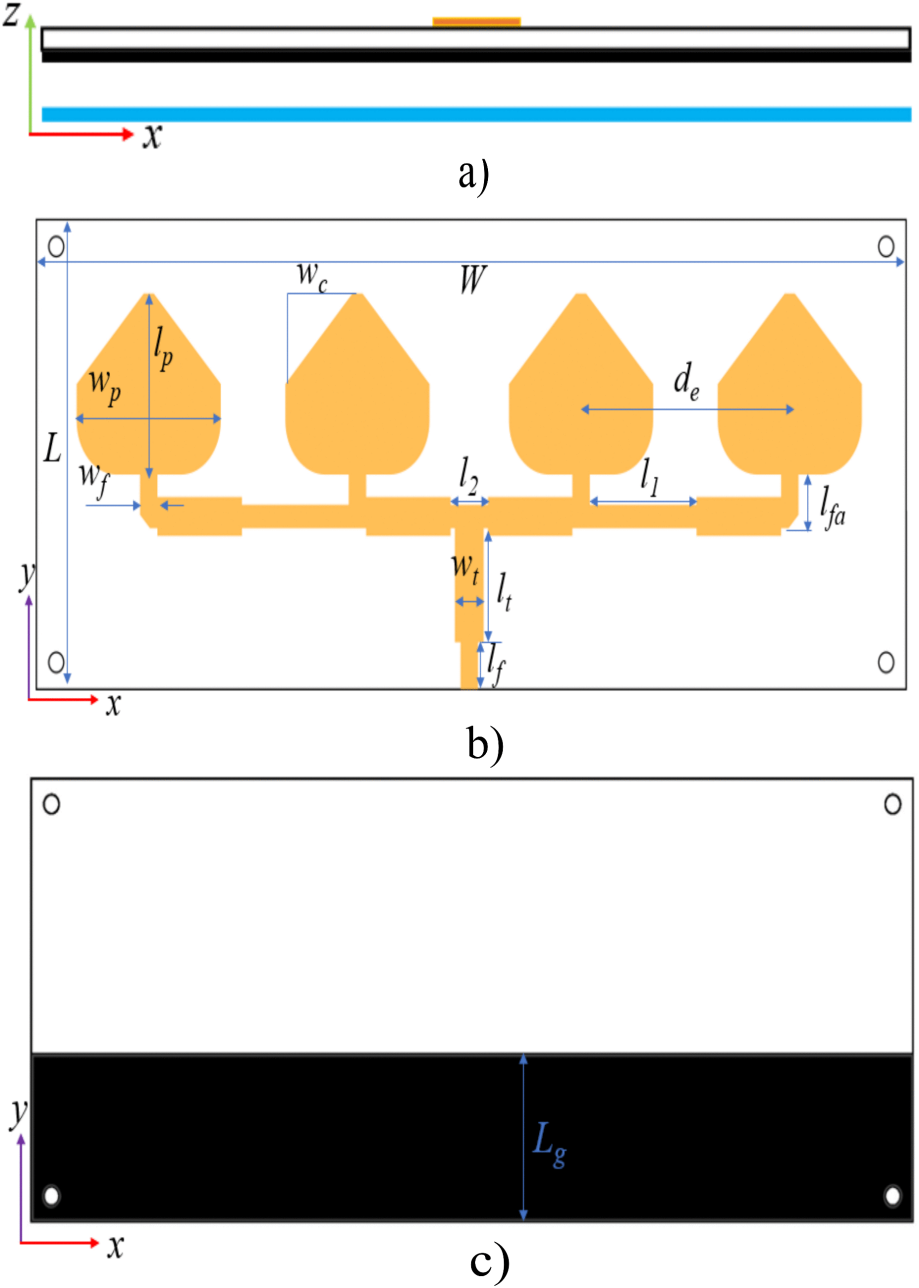
The model of the proposed array of 1 x 4: (a) cross-section view; (b) radiators; (c) partial ground layer. *W* = 175, *L* = 70, *w*_*p*_ = 29, *l*_*p*_ = 27, *w*_*c*_ = 13.5, *d*_*e*_ = 42, *l*_*1*_ = 21.5, *l*_*2*_ = 7.5, *w*_*f*_ = 3.5, *w*_*t*_ = 5.8, *l*_*t*_ = 17, *l*_*f*_ = 7 (unit: mm).


The detailed configurations of the proposed antenna are depicted in Fig 5, which consists of four elements arranged into an array of 1 x 4. Constructed on RO4350B substrate with a thickness of 1.524 mm, the antenna has an overall size of 175 × 70 × 7.324 mm^3^ (2.32λ x 0.93λ x 0.098λ). The dimensions of each element are 29 × 27 mm, and the separation between from center to center is 42 mm, corresponding to approximately 0.55λ. Impedance matching is achieved using λ/4 transformers, whose characteristic impedance is defined as:

$$Z_{T}=\frac{Z_{\text{0}}}{\sqrt{\text{2}}}$$ (1)

where Z_0_ is the characteristic impedance of the transmission line.

**Fig 6:**
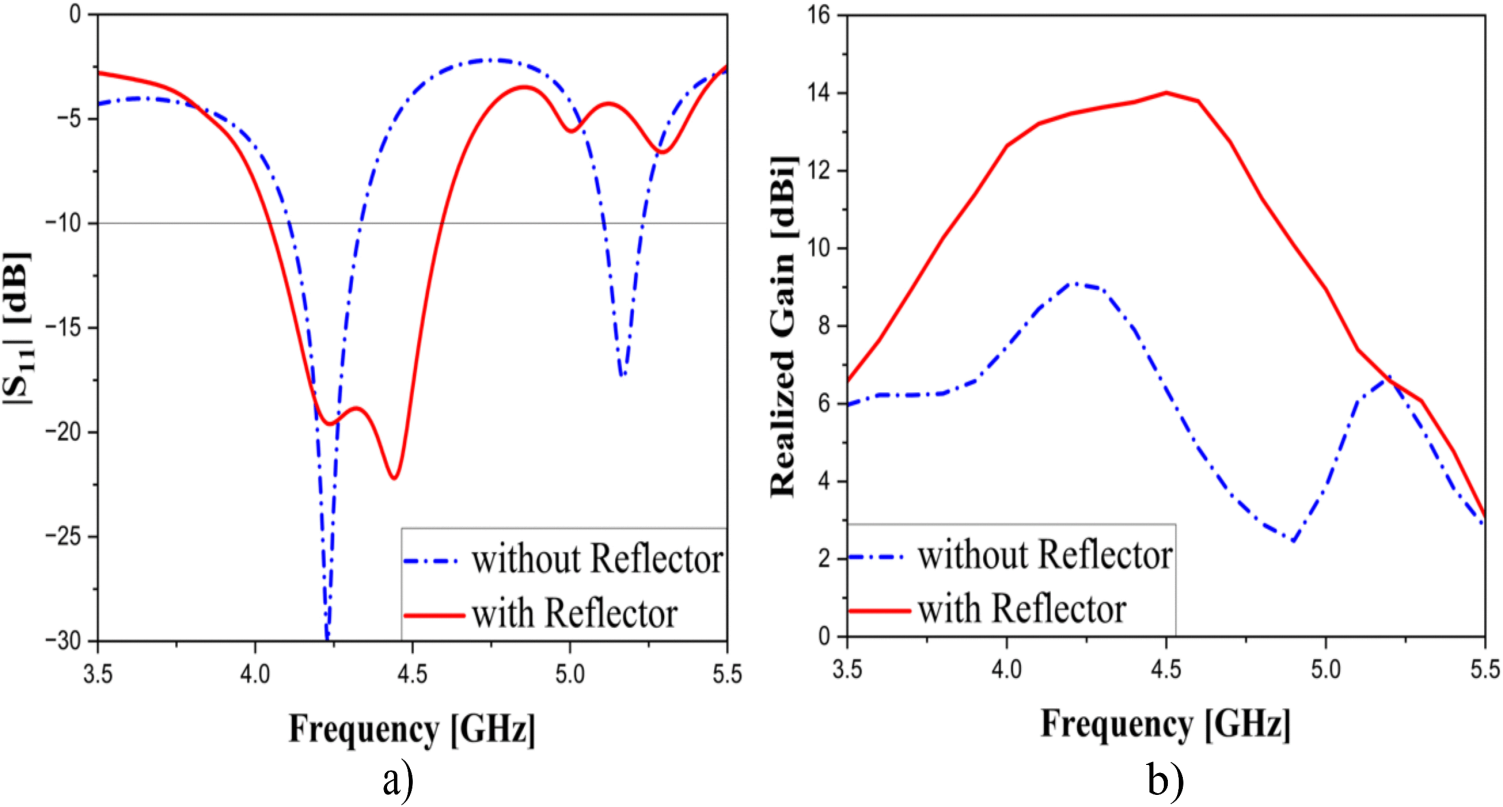
|S_11_| (left) and Realized Gain (right) with and without the second ground layer.

To enhance the antenna’s bandwidth, a partial ground measuring 175 × 26.5 mm is positioned opposite the radiation elements. Moreover, a reflector, which is a pure copper laminate, is utilized to enhance the antenna’s directivity and gain. To emphasize the role of the proposed reflector on antenna performance improvement, comparative simulations with and without it were carried out, and its results are illustrated in Fig 6. It is important to note that, for a fair comparison, the design without the reflector was optimized to achieve the best possible performance. As seen, although the impedance matching is deeper without a reflective surface, both the bandwidth and gain are inferior compared to when a reflective surface is used. Specifically, not only did the bandwidth and gain improve from 230 MHz (5.3%) and 9.1 dBi to 560 MHz (13%) and 14.01 dBi, respectively, but the antenna efficiency also increased due to impedance matching being ensured over a wider frequency range. When the reflector is introduced, the side-lobe and back-lobe levels are significantly reduced, so that more radiated energy is directed toward the main beam. This redistribution results for the observed improvement in antenna gain and directivity. Moreover, the presence of the reflector supports the excitation of multiple resonant modes. In this case, at least two consecutive resonances are observed, which contributes to a broader impedance bandwidth.

Moreover, the comparison in terms of performance between the proposed reflector and the AMC surface is implemented. The AMC surface consists of 11 x 5 unit-cells, in which the dimension of each unit cell is 10 x 10 mm and the separation between them is 14.5 mm.

**Fig 7:**
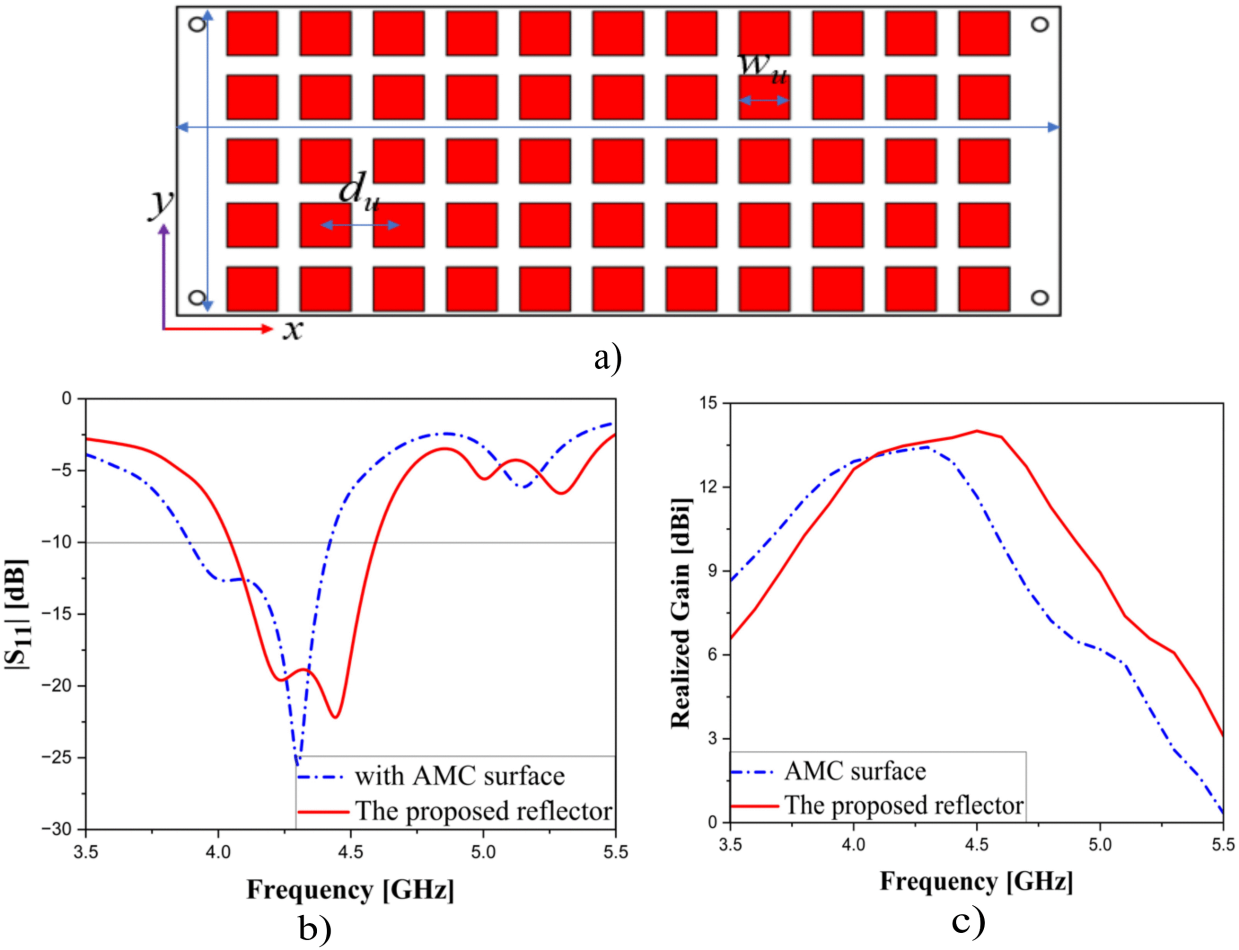
The model of AMC surface (a) and the performance-based comparison for using AMC surface and the proposed reflector: |S_11_| (b) and Realized Gain (c). *w*_*u*_ = 10, *d*_*u*_ = 14.5 (unit: mm).

Fig 7 provides a performance-based comparison between using the AMC surface and the proposed reflector to emphasize their differences. It should be noted that the dielectric layer used for the AMC surface is still 4350B with a thickness of 1.524 mm. As seen, not only does the proposed reflector exhibit a wider bandwidth of 560 MHz (4.04–4.6 GHz), but it also delivers a higher peak gain of 14.01 dBi, while for AMC surface, which reaches 530 MHz (3.89–4.42 GHz) and 13.43 dBi, respectively. In addition, the proposed reflector consistently maintains higher gain levels within the 4–4.6 GHz range (12.64–14.01 dBi), whereas the AMC surface yields lower values ranging from 10 to 13.43 dBi.

**Fig 8:**
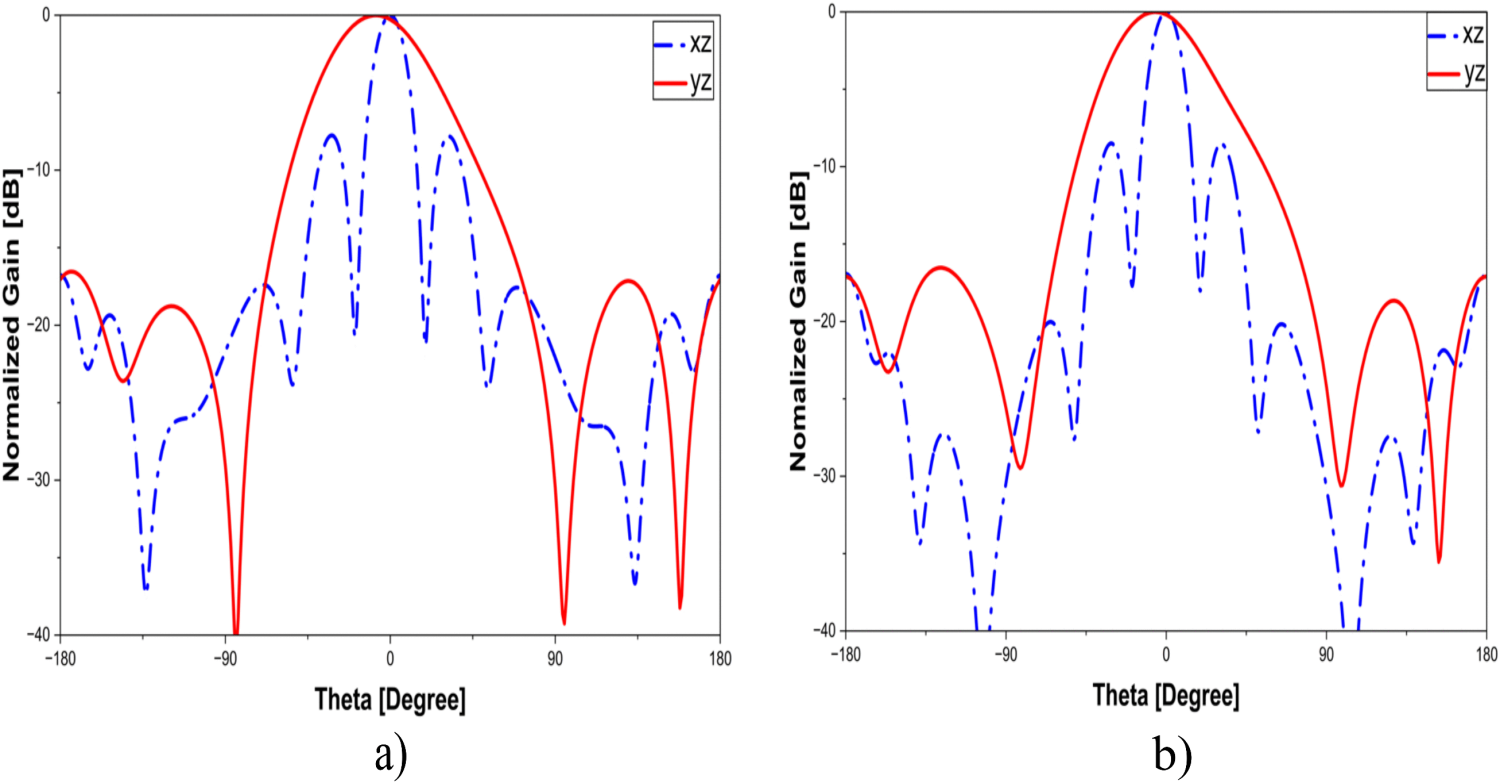
The side-lobe level of the proposed antenna in x-z and y-z planes: (a) 4.3 GHz, (b) 4.5 GHz.

**Fig 9:**
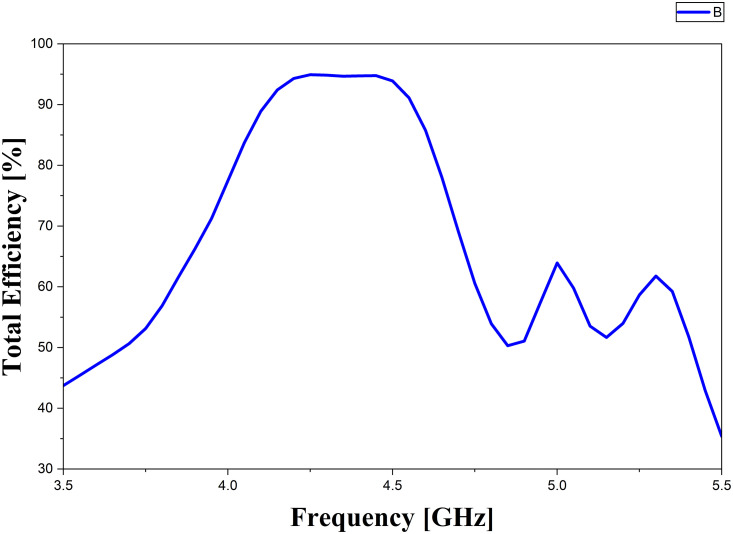
The total efficiency of the proposed antenna.

The sidelobe-level characteristics and the total efficiency of the proposed antenna are illustrated in Fig 8 and Fig 9, respectively. As illustrated, at 4.3 GHz, the SLLs in the x-z and y-z planes are measured to be −7.7 dB and −16.5 dB, respectively, while at 4.5 GHz they are −8.5 dB and −16.5 dB. The side-lobe level in the x-z plane is higher than that in the y-z plane due to unwanted radiations. As shown in Fig 8, the efficiency varies between 78% and 94% across the full operating frequency range of 4.04–4.6 GHz. Impedance matching plays a crucial role in determining the overall radiation efficiency of the antenna. In the operating band from 4.18 to 4.5 GHz, where the reflection coefficient remains below −18 dB, the antenna achieves a radiation efficiency higher than 94%, clearly illustrating the relationship between proper impedance matching and efficiency.

### Parametric studies

**Fig 10:**
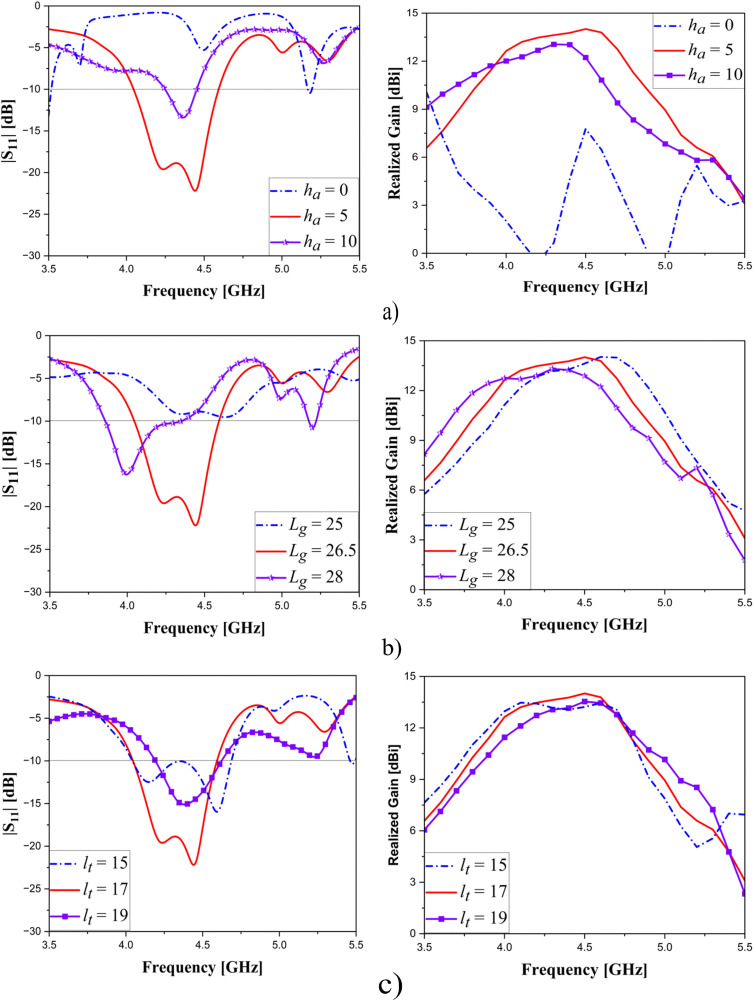
Variation analysis of the parameters:(a) air gap (*h*_*a*_); (b) the length of partial ground (*L*_*g*_); (c) the length of transformer (*l*_*t*_).


Parametric analysis of important parameters is implemented to achieve optimal antenna performance, and these investigations, including parameters: the height of air gap (*h*_*a*_); the length of partial ground (*L*_*g*_), and the length of transformer (*l*_*t*_) are displayed in Fig 10. For each scenario, only one parameter is varied, with all others kept unchanged. According to Fig 10(a), for the cases of *h*_*a*_ = 0 mm and *h*_*a*_ = 10 mm, the phase of the reflector is not aligned with that of the antenna, which leads to destructive interference and consequently degrades the overall performance. In contrast, when *h*_*a*_ = 5 mm, the reflected wave remains in phase with the antenna’s radiation, enabling constructive interference and resulting in enhanced efficiency. Meanwhile, varying the length of the partial ground (*L*_*g*_) is shown in Fig 10(b). We know that the resonant frequency is given [29]:

$$f_{r}=\frac{\text{1}}{\text{2}\pi \sqrt{L_{eq}C_{eq}}}$$ (2)

Therefore, a larger *L*_*g*_ introduces more capacitance and inductance, pushing the resonance to a lower frequency. When *L*_*g*_ is shorter, these parasitic values are reduced and the resonance moves upward. At *L*_*g*_ = 25 mm, although a resonance occurs, the impedance does not match well with 50 Ω, likely due to a phase mismatch between the ground current and the feed excitation. Considering both |S_11_| and gain, *L*_*g*_ = 26.5 mm provides the best compromise and therefore, this value is selected. Fig 10(c) exhibits the influence of the transformer length (*l*_*t*_) to the |S_11_| and gain. As aforementioned, the transformer length (*l*_*t*_) is utilized to match impedance for antenna. Increasing *l*_*t*_ initially improves the matching due to better phase compensation. However, when *l*_*t*_ exceeds the optimal electrical length, the transformer introduces excessive phase shift, causing the resonance to move toward higher frequencies instead of lowering. Therefore, the matching is the best with *l*_*t*_ = 17 mm. The performance is significantly declined when *l*_*t*_ = 15 mm and *l*_*t*_ = 19 mm. Finally, the chosen parameters for scenarios are *h*_*a*_ = 5 mm, *L*_*g*_ = 26.5 mm and *l*_*t*_ = 17 mm.

## Experimental results and discussion

**Fig 11:**
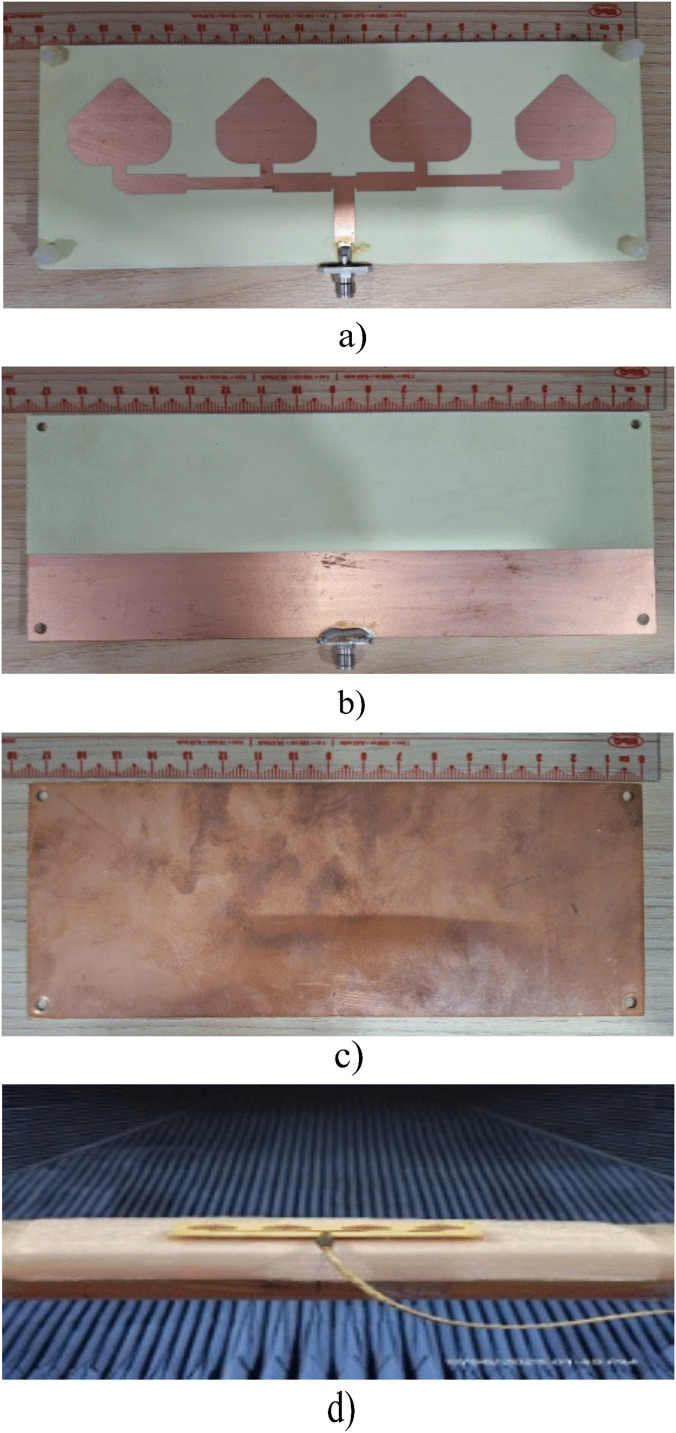
The photograph of the proposed antenna: (a) radiation elements; (b) partial ground; (c) copper laminate; (d) measurement setup.

**Fig 12:**
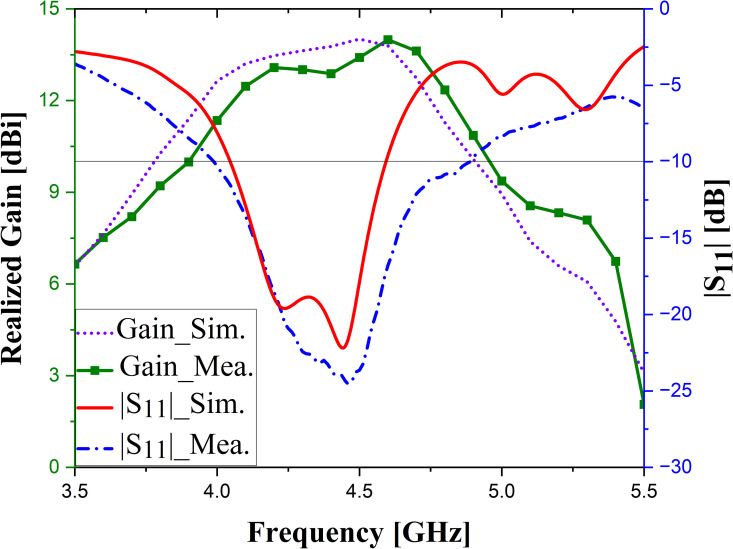
The simulated and measured results of the fabricated prototype.

**Fig 13:**
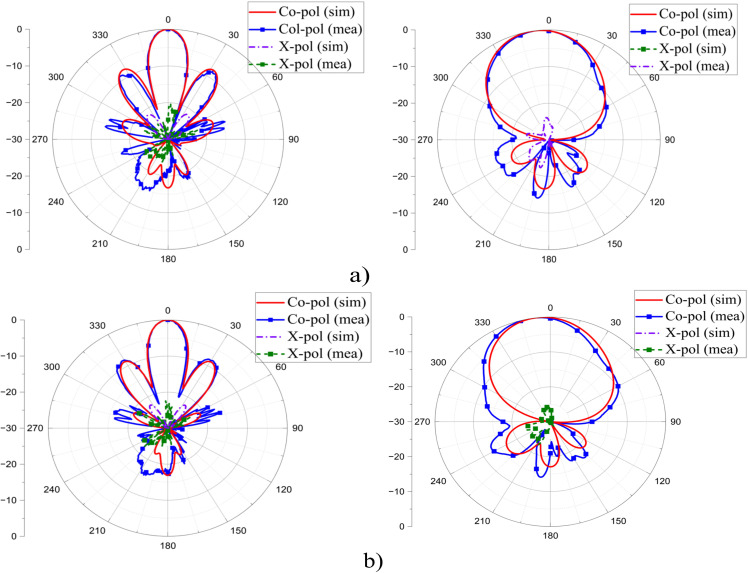
Radiation pattern in x-z (left) and y-z (right) planes: (a) 4.3 GHz, (b) 4.5 GHz.

To validate the design concept, measurements are conducted on a fabricated antenna prototype, as illustrated in Fig 11. The proposed antenna comprises four patch elements with a power divider and the partial ground printed on two sides of the RO4350B dielectric layer and a pure copper laminate. In addition, plastic screws are used to hold the dielectric and copper laminate layers in place, ensuring the most accurate manufacturing possible. S-parameter is measured using a Keysight E5071C network analyzer under open-air conditions, while far-field radiation characteristics are tested in an anechoic chamber. Fig 12 shows the simulated and measured S-parameter and realized gain of the proposed design, whereas simulation and measurement data of the radiation pattern are presented in Fig 13. As observed, the simulation and measurement results agree rather closely. The measured and simulated data indicate that the proposed design achieves 10-dB impedance bandwidth (IBW) are from 3.98 to 4.9 GHz and from 4.04 to 4.6 GHz, corresponding to 21.4% and 13%, respectively. In addition, within the 3.9–4.9 GHz range, the gain values fluctuate from 10.1–14.01 dBi for simulation and 10.9–14 dBi for measurement. Slight discrepancies are attributed to imperfections in the measurement chamber. Several secondary factors may also contribute, including (i) the weld and losses caused by the SMA connector, (ii) dielectric and copper losses in the substrate, and (iii) tolerances from fabrication progress. In addition, the RF feed cable remaining in the chamber during testing (as shown in Fig 8(d)) may also contribute to these variations. Nevertheless, the overall agreement between measured and simulated data remains consistent, with differences falling within acceptable margins.

To validate the effectiveness of the proposed approach, a summary of the comparison between this work and recently reported antennas is provided in Table 1. It is evident that most existing designs exhibit lower gain compared to the proposed antenna, except for the designs reported in [31,39,41]. The antennas reported in [31,39] comprise 8 and 16 elements, while the proposal in [41] is designed at the higher frequency (13 GHz). Therefore, it is reasonable that the gain values of these antennas are higher than those of the proposed design. However, a notable drawback of the designs in [31,39,41] is its large physical size, and this is also observed in the proposals of [32,36,37] while the antennas in [30,33,34,37,38] suffer the narrow bandwidth (under 15%). Meanwhile, the low gain is the limit of the designs in [35,40,42]. Moreover, the designs in [31,32,34,37,38] have high complexity when using the techniques of high density interconnect, substrate integrated waveguide (SIW), fractal, microstrip-to-waveguide transition, and these lead to difficulty for fabrication as well as increased production costs. In comparison, with only four elements, the proposed antenna achieves wide bandwidth, high efficiency, and high gain. These promising characteristics highlight its potential for further research and practical applications in satellite communication systems.

**Table 1:** The comparison between this work and recently reported antennas.

Antenna/ Year	Design Methodology	Complexity	Substrate/$\epsilon_{r}$	Frequency [GHz]	Array size	Bandwidth [%]	Peak gain [dBi]	Total Efficiency [%]	Size [λ^3^]
[30]/ 2020	Series feeding, two substrate layers	Low	RO3206, TLY5/6.15, 2.18	2.37	4	1.8	9.8	N/G	N/G
[31]**/** 2021	High density interconnect, two substrate layers	High	HL-972-LF, GHPL-970LF/3.4	25.3	8	20.6	14.2	N/G	2.3 × 2
[32]/ 2021	SIW, a substrate layer	High	FR4/4.5	12.2	10	35	8	< 50	7.2 × 0.8 × 0.06
[33]/ 2021	Series inclined slot-feeding	Low	RO4003C/3.38	28	4	10.9	11.65	N/G	0.94 × 2.6 × 0.047
[34]/ 2022	Substrate integrated suspended line, five substrate layers	High	FR4/ N/G	3.5	2	14.4	11.14	> 75	1.3 × 1 × 0.05
[35]/ 2022	Metasurface, two substrate layers	Low	RO4003/3.38	1.8	4	40	8.7	N/G	0.67 × 0.67 × 0.06
[36]**/** 2023	Metasurface, two substrate layers	Low	RO4003/3.38	28.5	4	21.5	11	N/G	2.86 × 2.86 × 2.78
[37]/ 2024	SIW, fractal, a substrate layer	High	RT5880/2.2	8.15	4	14.8	12.5	89	4.8 × 1.32 × 0.086
[38]/ 2024	Microstrip-to-waveguide transition, a substrate layer	High	RO4830/0.125	63.8	3	7.7	11.06	> 75	0.283 × 1.412 × 0.027
[39]/ 2024	Metasurface, two substrate layers	Low	F4BM/2.65	5.3	16	17	14.9	97	1.6 × 1.6 × N/G
[40]/ 2025	Metasurface, two substrate layers	Low	RO4003/3.38	0.9	4	8	7.7	N/G	0.6 × 0.6 × 0.09
[41]/ 2025	Conductive element, two substrate layers	Low	RO4003/3.38	13	4	20	14.55	N/G	5.05 × 1.51 × 0.28
[42]/ 2025	Wilkinson power divider, a substrate layer	Low	FR4/4.4	3.5	8	6	9.3	58.9	2.91 x 0.81 x 0.018
**This work**	**Reflector**	**Low**	**RO4350B/3.66**	**4.3**	**4**	**21.4**	**14**	**> 80**	**2.32 x 0.93 x 0.098**

## Conclusion

This work investigates an antenna array consisting of four linearly arranged patch elements (1 × 4). This study demonstrates that incorporating a pure copper laminate as a reflector remarkably enhances the antenna’s gain (approximately 5 dB) while the adoption of a partial ground structure contributes to broaden the bandwidth. The manufactured prototype achieves a 10-dB impedance bandwidth of 21.4% (3.98–4.9 GHz), a peak gain of 14 dBi, and maintains a high radiation efficiency of 80% within the operating band. Notably, the employed design strategies in this paper ensure simplicity, minimizing complexity for fabrication and optimization. The proposed approach can also be combined with other techniques to further improve antenna performance.

## References

[1] Li M, Su Y, Yao Y, Zhang W, Lin X, Fan Y. A Broadband Dual-Polarized C-/Ku-Band Shared Aperture Antenna Array for Satellite Communications. IEEE Trans Antennas Propagat. 2024;72(11):8840–5. doi: 10.1109/tap.2024.3439827

[2] Koli MNY, Afzal MU, Esselle KP, Hashmi RM. An All-Metal High-Gain Radial-Line Slot-Array Antenna for Low-Cost Satellite Communication Systems. IEEE Access. 2020;8:139422–32. doi: 10.1109/access.2020.3012787

[3] Li X, Ma R, Cai H, Pan Y-M, Zhang XY. High-Gain Dual-Band Aperture-Shared CP Patch Antenna With Wide AR Beamwidth for Satellite Navigation System. Antennas Wirel Propag Lett. 2023;22(8):1888–91. doi: 10.1109/lawp.2023.3268653

[4] Nawaz H, Niazi AU, Ahmad M. Dual Circularly Polarized Patch Antenna with Improved Interport Isolation for S-Band Satellite Communication. Int J Antennas Propag. 2021;2021:1–10. doi: 10.1155/2021/8022207

[5] Xie L, Chi L, Lin B, Wang X, Qi Y. A compact channel patch antenna with reconfigurable circularly polarized pattern for mobile satellite communications. AEU - Int J Electron Commun. 2023;164:154632. doi: 10.1016/j.aeue.2023.154632

[6] El-Din MSHS, Shams SI, Allam AMMA, Gaafar A, Elhennawy HM, Fathy Abo Sree M. SIGW Based MIMO Antenna for Satellite Down-Link Applications. IEEE Access. 2022;10:35965–76. doi: 10.1109/access.2022.3160473

[7] Jin H, Luo GQ, Wang W, Che W, Chin K-S. Integration Design of Millimeter-Wave Filtering Patch Antenna Array With SIW Four-Way Anti-Phase Filtering Power Divider. IEEE Access. 2019;7:49804–12. doi: 10.1109/access.2019.2909771

[8] Elahi M, Trinh-Van S, Yang Y, Lee K-Y, Hwang K-C. Compact and High Gain 4 × 4 Circularly Polarized Microstrip Patch Antenna Array for Next Generation Small Satellite. Appl Sci. 2021;11(19):8869. doi: 10.3390/app11198869

[9] Wang X-Y, Tang S-C, Yang L-L, Chen J-X. Differential-Fed Dual-Polarized Dielectric Patch Antenna With Gain Enhancement Based on Higher Order Modes. IEEE Antennas Wirel Propag Lett. 2020;19(3):502–6. doi: 10.1109/lawp.2020.2964569

[10] Awais M, Madni A, Khan WT. Design of a Compact High Isolation 4-Element Wideband Patch Antenna Array for GNSS Applications. IEEE Access. 2022;10:13780–6. doi: 10.1109/access.2022.3147600

[11] Jin X, Qiu Y, Wu D, Yu G, Guo R, Wu G, et al. A Low-Profile Dual-Polarized MIMO Antenna with an AMC Surface for WLAN Applications. Int J Antennas Propag. 2021;2021:1–12. doi: 10.1155/2021/9218255

[12] Dicandia FA, Genovesi S. Characteristic Modes Analysis of Non-Uniform Metasurface Superstrate for Nanosatellite Antenna Design. IEEE Access. 2020;8:176050–61. doi: 10.1109/access.2020.3027251

[13] Gao P, Xu F. A Low-Profile Broadband Circularly Polarized Pattern Diversity Metasurface-Inspired Antenna. IEEE Trans Antennas Propagat. 2023;71(10):8308–13. doi: 10.1109/tap.2023.3295116

[14] Jiang H, Yan N, Ma K, Wang Y. A Wideband Circularly Polarized Dielectric Patch Antenna With a Modified Air Cavity for Wi-Fi 6 and Wi-Fi 6E Applications. IEEE Antennas Wirel Propag Lett. 2023;22(1):213–7. doi: 10.1109/lawp.2022.3201077

[15] Astuti DW, Fadilah R, Muslim M, Rusdiyanto D, Alam S, Wahyu Y. Bandwidth Enhancement of Bow-tie Microstrip Patch Antenna Using Defected Ground Structure for 5G. J Commun. 2022;:995–1002. doi: 10.12720/jcm.17.12.995-1002

[16] Jung J-I, Yang J-R. 5.8-GHz Patch Antenna with an Enhanced Defected Ground Structure for Size Reduction and Increased Bandwidth. J Electromagn Eng Sci. 2022;22(3):245–51. doi: 10.26866/jees.2022.3.r.83

[17] Meng F, Liu Y, Sharma SK. A miniaturized patch antenna with enhanced bandwidth by using reactive impedance surface ground and coplanar parasitic patches. Int J RF Microw Comput Aided Eng. 2020;30(7):1–10. doi: 10.1002/mmce.22225

[18] Amillia F, Setijadi E, Hendrantoro G. The Effect of Parasitic Patches Addition on Bandwidth Enhancement and Mutual Coupling in 2 × 2 Sub-Arrays. IEEE Access. 2022;10:72057–64. doi: 10.1109/access.2022.3185999

[19] AboEl-Hassan M, Farahat AE, Hussein KFA. Wideband star-shaped antenna based on artificial magnetic conductor surface for unidirectional radiation. AEU - Int J Electron Commun. 2025;189:155603. doi: 10.1016/j.aeue.2024.155603

[20] Wang W, Jin H, Yu W, Zhang XH, Liu L, Chin K-S, et al. Design of a High-Order 2 × 2 SIW Cavity-Backed Patch Subarray and Its Application in Millimeter-Wave CP Filtenna Array. IEEE Antennas Wirel Propag Lett. 2023;22(4):739–43. doi: 10.1109/lawp.2022.3223932

[21] Circularly AB, Patch P, Array A, Oblique W. A Broadband Circularly Polarized Patch Antenna Array With Oblique Beam for Satellite Wide Scanning Coverage Applications. IEEE Trans Antennas Propag. 2025;73:3388–93. doi: 10.1109/TAP.2025.3529192

[22] Karami F, Rezaei P, Amn‐e‐Elahi A, Abolfathi A, A. Kishk A. Broadband and efficient patch array antenna fed by substrate integrated waveguide feed network for Ku‐band satellite applications. Int J RF Microw Comput Aided Eng. 2021;31(9). doi: 10.1002/mmce.22772

[23] Malleswari NSN, Sarita V, Archana S, Deep BK, Mohith I. Y-Patch MIMO Antenna Design for Dual-Band Wireless Applications. In: 2024 International Conference on Advances in Modern Age Technologies for Health and Engineering Science (AMATHE). 2024. p. 1–5. doi: 10.1109/amathe61652.2024.10582084

[24] Nguyen NL, Vu VY. Gain enhancement for MIMO antenna using metamaterial structure. Int J Microw Wireless Technol. 2019;11(08):851–62. doi: 10.1017/s175907871900059x

[25] Biswas M, Dam M, Banik S. Theoretical and experimental investigation of two-layer circular patch antenna excited by a coaxial probe. Int J Microw Wireless Technol. 2021;14(1):95–114. doi: 10.1017/s1759078721000222

[26] Anandkumar D, Sangeetha RG. Design and analysis of aperture coupled micro strip patch antenna for radar applications. Int J Intell Netwrk. 2020;1:141–7. doi: 10.1016/j.ijin.2020.11.002

[27] Grilo M, Correra FS. Rectangular patch antenna on textile substrate fed by proximity coupling. J Microwaves, Optoelectron Electromagn Appl. 2015;14:SI103–12.

[28] Peram A, Subba Rami Reddy A, Giri Prasad MN. Miniaturized Single Layer Ultra Wide Band (UWB) Patch Antenna Using a Partial Ground Plane. Wireless Pers Commun. 2019;106(3):1275–91. doi: 10.1007/s11277-019-06213-4

[29] Pozar M. Microwave engineering. 4 edition. John Wiley & Sons, Inc.; 2012.

[30] Hilario Re PD, Comite D, Podilchak SK. Single-Layer Series-Fed Planar Array With Controlled Aperture Distribution for Circularly Polarized Radiation. IEEE Trans Antennas Propagat. 2020;68(6):4973–8. doi: 10.1109/tap.2019.2952001

[31] Wang L, Shi J, Xu K, Yin ZW. Compact Dual-Strip Coupled Dual-Patch Antenna for Millimeter-Wave AiP Applications. IEEE Antennas Wirel Propag Lett. 2021;20(4):577–81. doi: 10.1109/lawp.2021.3057430

[32] Eid AM, Salama AA, Elkamchouchi HM. A novel circularly polarized slotted substrate integrated waveguide antenna array for satellite applications. IET Microwaves Antenna Prop. 2021;15(8):925–36. doi: 10.1049/mia2.12100

[33] Ullah U, Al-Hasan M, Koziel S, Mabrouk IB. A Series Inclined Slot-Fed Circularly Polarized Antenna for 5G 28 GHz Applications. IEEE Antennas Wirel Propag Lett. 2021;20(3):351–5. doi: 10.1109/lawp.2021.3049901

[34] Wang T, Yan N, Tian M, Luo Y, Ma K. A Low-Cost High-Gain Filtering Patch Antenna With Enhanced Frequency Selectivity Based on SISL for 5G Application. Antennas Wirel Propag Lett. 2022;21(9):1772–6. doi: 10.1109/lawp.2022.3179522

[35] Cui J, Zhao X, Sheng W. Low profile and broadband circularly polarized metasurface antenna based on nonuniform array. AEU - Int J Electron Commun. 2022;156:154386. doi: 10.1016/j.aeue.2022.154386

[36] Nkimbeng CHS, Wang H, Byun G, Park YB, Park I. Non-Uniform Metasurface-Integrated Circularly Polarized End-Fire Dipole Array Antenna. J Electromagn Eng Sci. 2023;23(2):109–21. doi: 10.26866/jees.2023.2.r.150

[37] Rabie MM, El-Gendy MS, El Damak AR, Ibrahim F, El-Hennawy H. Circularly Polarized Double-walled SIW Fractal Slot and Hexagonal Ring Slot Antenna Array for X-band Satellite Applications. PIER B. 2024;105:1–15. doi: 10.2528/pierb24010402

[38] Lee J, Park S, Choi J, Park W, Jung KY. Compact series-fed microstrip patch array antenna in the 60 GHz band. AEU - Int J Electron Commun. 2024;187:155513. doi: 10.1016/j.aeue.2024.155513

[39] Zhu L, Sun J, Xu G, Hao Z, Cao Q. An Integrated Antenna Array With Broadband, Low-RCS, and High-Gain Characteristics. IEEE Trans Antennas Propagat. 2024;72(6):5408–13. doi: 10.1109/tap.2024.3396658

[40] Ngoc Hien Doan T, Dang Le H, Son Hoang van, Nguyen KK, Xuat Ta S. A Low-Profile Broadband Circularly Polarized Slot Antenna Loaded With Metasurface for UHF-RFID Readers. IEEE Access. 2025;13:93057–62. doi: 10.1109/access.2025.3574117

[41] Teimouri MH, Ghobadi C, Nourinia J. A Ku-Band dual-sense CP array antenna with polarization converter element. AEU - Int J Electron Commun. 2025;190:155637. doi: 10.1016/j.aeue.2024.155637

[42] Xiao W, Fan K, Zhou F, Zhu J, Li S. A 3.45 GHz linear array antenna based on Wilkinson power divider structure. AEU - Int J Electron Commun. 2025;189:155594. doi: 10.1016/j.aeue.2024.155594

